# Variable Processing and Cross-presentation of HIV by Dendritic Cells and Macrophages Shapes CTL Immunodominance and Immune Escape

**DOI:** 10.1371/journal.ppat.1004725

**Published:** 2015-03-17

**Authors:** Jens Dinter, Ellen Duong, Nicole Y. Lai, Matthew J. Berberich, Georgio Kourjian, Edith Bracho-Sanchez, Duong Chu, Hang Su, Shao Chong Zhang, Sylvie Le Gall

**Affiliations:** Ragon Institute of MGH, MIT and Harvard, Massachusetts General Hospital, Harvard Medical School, Cambridge, Massachusetts, United States of America; University of Wisconsin, UNITED STATES

## Abstract

Dendritic cells (DCs) and macrophages (Møs) internalize and process exogenous HIV-derived antigens for cross-presentation by MHC-I to cytotoxic CD8^+^ T cells (CTL). However, how degradation patterns of HIV antigens in the cross-presentation pathways affect immunodominance and immune escape is poorly defined. Here, we studied the processing and cross-presentation of dominant and subdominant HIV-1 Gag-derived epitopes and HLA-restricted mutants by monocyte-derived DCs and Møs. The cross-presentation of HIV proteins by both DCs and Møs led to higher CTL responses specific for immunodominant epitopes. The low CTL responses to subdominant epitopes were increased by pretreatment of target cells with peptidase inhibitors, suggestive of higher intracellular degradation of the corresponding peptides. Using DC and Mø cell extracts as a source of cytosolic, endosomal or lysosomal proteases to degrade long HIV peptides, we identified by mass spectrometry cell-specific and compartment-specific degradation patterns, which favored the production of peptides containing immunodominant epitopes in all compartments. The intracellular stability of optimal HIV-1 epitopes prior to loading onto MHC was highly variable and sequence-dependent in all compartments, and followed CTL hierarchy with immunodominant epitopes presenting higher stability rates. Common HLA-associated mutations in a dominant epitope appearing during acute HIV infection modified the degradation patterns of long HIV peptides, reduced intracellular stability and epitope production in cross-presentation-competent cell compartments, showing that impaired epitope production in the cross-presentation pathway contributes to immune escape. These findings highlight the contribution of degradation patterns in the cross-presentation pathway to HIV immunodominance and provide the first demonstration of immune escape affecting epitope cross-presentation.

## Author Summary

Pathogens such as HIV can enter cells by fusion at the plasma membrane for delivery in the cytosol, or by internalization in endolysosomal vesicles. Pathogens can be degraded in these various compartments into peptides (epitopes) displayed at the cell surface by MHC-I. The presentation of pathogen-derived peptides triggers the activation of T cell immune responses and the clearance of infected cells. How the diversity of compartments in which HIV traffics combined with the diversity of HIV sequences affects the degradation of HIV and the recognition of infected cells by immune cells is not understood. We compared the degradation of HIV proteins in subcellular compartments of dendritic cells and macrophages, two cell types targeted by HIV and the subsequent presentation of epitopes to T cells. We show variable degradation patterns of HIV according to compartments, and the preferential production and superior intracellular stability of immunodominant epitopes corresponding to stronger T cell responses. Frequent mutations in immunodominant epitopes during acute infection resulted in decreased production and intracellular stability of these epitopes. Together these results demonstrate the importance of protein degradation patterns in shaping immunodominant epitopes and the contribution of impaired epitope production in all cellular compartments to immune escape during HIV infection.

## Introduction

Cytotoxic CD8^+^ T cell (CTL) responses play an important role in the outcome of viral infections. CTL responses elicited during HIV or HCV infection follow a predictable immunodominance hierarchy, whereby immunodominant T cell responses are defined by a higher frequency in a population sharing a HLA, or by a higher magnitude of interferon-gamma production in an individual [1]. The acute phase of HIV infection is characterized by narrow immunodominance patterns [2,3], and immune pressure leading to frequent escape mutations in immunodominant epitopes changes the T cell response hierarchy during disease progression [4–9]. Since immunodominance established during HIV infection or reproduced by some HIV vaccines does not clear or prevent infection, breaking immunodominance hierarchies to induce the presentation of broader subdominant but protective epitopes provides an interesting alternative for vaccine design.

Immunodominance is shaped by multiple factors [10], including binding affinity to MHC or the TCR [11,12], frequency of CD8^+^ T cell precursors and the TCR repertoire [13], kinetics of expression and amount of viral proteins [14], and efficiency of antigen processing [15–17]. How degradation patterns during cross-presentation of antigens, specifically in the case of highly variable pathogens like HIV, may shape immunodominance and viral evolution is not well understood. Antigen presenting cells (APC) such as DCs and Møs cross-present antigens from various sources, such as cell-associated antigens [18–21], viral particles [22–24], or viral proteins [25,26] for priming or activation of T cell responses. Internalized antigens first undergo proteolytic processing by cathepsins in endocytic compartments [27] where they can be loaded onto MHC I or MHC II molecules for presentation to CD8^+^ or CD4^+^ T cells [28], or eventually escape into the cytosol [29] for additional degradation [30,31], translocation in the ER and cross-presentation by MHC I.

The cell type and the trafficking of antigens have a crucial impact on their processing, as different proteases in each compartment can produce or destroy epitopes, thus shaping the surface peptidome [25,32,33]. Different cell types express individual patterns of proteases, which affect epitope processing as we previously showed for the degradation of several HIV epitopes by cytosolic peptidases [34,35]. In a given cell type, the degradation of proteins in the cytosol and in the ER [34–36] contribute to defining the timing and amount of peptides available for presentation, and have been shown to preferentially produce multiple epitopes corresponding to immunodominant responses in HIV [16,17] and HCV infection [15]. Moreover, differences in degradation patterns of HIV peptides in cytosolic, endosomal or lysosomal cell extracts isolated from human PBMCs [37,38] further highlight the critical role of antigen trafficking on epitope processing. Mutations within and outside epitopes alter degradation patterns by proteasomes and aminopeptidases in the cytosol or in the ER, reduce epitope presentation and lead to immune escape [39–42]. Nothing is known about the impact of these mutations on the degradation patterns during cross-presentation despite its potential impact for T cell priming and activation.

The aim of this study was to systematically examine the processing and cross-presentation of dominant and subdominant HIV Gag-derived epitopes and of natural mutants of an immunodominant epitope by monocyte-derived DCs and Møs. We showed a preferential production and a superior intracellular stability of peptides containing immunodominant epitopes in cytosol and endolysosomes. Moreover, we showed that frequent HLA-restricted mutations in an immunodominant peptide associated with shifts in immunodominance patterns, modified the degradation patterns of HIV fragments in endolysosomes and reduced epitope stability and production in the cross-presentation pathway. These results highlight the contribution of degradation patterns in the cross-presentation pathways of APC to immunodominance and immune escape in HIV infection.

## Materials and Methods

### Ethics statement

Cells were isolated from HLA-typed blood donors or anonymous buffy coats after written informed consent and approval by the Partners Human Research Committee under protocol 2005P001218 (Boston, USA).

### Cell culture

Human peripheral blood mononuclear cells (PBMCs) were isolated by Ficoll-Hypaque (Sigma-Aldrich) density centrifugation. Monocytes were enriched using CD14+ magnetic isolation kits (StemCell) and differentiated into DCs and Møs during a 6-day culture. DCs were cultured in AIM-V media with 1% human serum AB (Gemini Bio-Products) supplemented with 20ng/mL IL-4 and 10ng/mL GM-CSF (CellGenix). On days 2 and 4, fresh IL-4 and GM-CSF were added. Møs were cultured in ultra low attachment plates (Corning) in AIM-V media with 10% human serum AB. Where indicated, maturation of DCs and Møs was induced by TLR ligand stimulation with 2μg/mL LPS, 1μg/mL CL097, or 1μg/mL R848 (Invivogen) for 2 days [35]. Epitope-specific CTL clones were maintained in the presence of 50U/mL IL-2, using 0.1μg/mL CD3-specific mAb 12F6, and irradiated feeder cells as stimulus for T cell proliferation.

### ELISPOT cross-presentation assay

Immature DCs and Møs were exposed to recombinant HIV-1 p24-protein, HIV-1 p55-protein or control protein (Protein Sciences Corporation, USA) for 1hr at 37°C. Where indicated, cells were pre-incubated for 45 minutes with inhibitors for proteasome (10μM MG132 (Enzo Life Sciences)) or cysteine proteases (5μM E64 (Sigma-Aldrich)). Cells pulsed with equivalent molar concentrations of the optimal epitopes were used as controls for antigen presentation and CTL clone specificity. DCs and Møs were thoroughly washed and cultured overnight with epitope-specific CTL clones at a 2:1 effector-to-target ratio in 96-well plates (Millipore) coated with anti-IFN-y mAb 1-D1K (Mabtech). ELISPOT plates were washed and developed as described previously [43].

### P24 uptake assay

DCs or Møs (1x10^6^ cells/mL) were exposed to the following protease inhibitors for 45 minutes: 10μM MG132, 5μM E64, 10μM cathepsin S inhibitor Z-FL-COCHO, 10μM leupeptin (Enzo Life Sciences), 120μM bestatin (Sigma-Aldrich), before incubation with different concentrations of recombinant HIV-1 p24-protein for 1hr at 37°C or 4°C. Samples were thoroughly washed in ice-cold PBS and immediately treated with 3mg/mL pronase E (Sigma-Aldrich) in AIM-V media without serum for 10 minutes on ice. Cells were washed, lysed in 0.5% Triton X-100 containing lysis buffer and the amount of p24 protein in cell lysates was measured using a standard HIV-1 p24 antigen ELISA (Perkin Elmer).

### Fluorescent measurement of proteolytic activities in live cells and cell extracts

Whole cell extracts from DCs and Møs were prepared by 0.125% digitonin permeabilization in ice-cold lysis buffer (50mM HEPES, 50mM potassium acetate, 5mM MgCl_2_, 1mM DTT, 1mM ATP, 0.5mM EDTA, 10% Glycerol, pH 7.4), followed by 17,762 rcf centrifugation at 4°C for 15 minutes to remove cell debris as previously done [17,35,44]. The proteolytic activities of cathepsin S (cell, 50μM; extracts, 10μM Z-VVR-AMC), omni cathepsins (cell, 50μM; extracts, 50μM Z-FR-AMC), cathepsin D&E (extracts, 10μM Mca-GKPILFFRLK-Dnp, Enzo Life Sciences), and cathepsin B (extracts, 50μM Z-RR-AMC, Bachem) were measured by cleavage of peptide-specific fluorogenic substrates. Incubation with the relevant inhibitor of cathepsin S (10μM ZFL-COCHOO, Calbiochem), cathepsin B (10μM Z-RLVazaglyIV-OMe, Bachem), omnicathepsins (50μM E64), and cathepsin D&E (100μM Pepstatin A, Enzo Life Sciences) confirmed the specificity of reactions. For cells, 2x10^4^ DCs or Møs in PBS/0.0025% digitonin were used to measure the proteolytic activities. For cell extracts, equivalent amounts as determined by total protein concentration were used in reaction buffer (50mM sodium chloride, 50mM potassium phosphate, 2mM DTT, 2mM EDTA; pH 7.4, pH5.5 or pH4.0, respectively). The rate of fluorescence emission, which is proportional to the proteolytic activity, was measured every 5 minutes at 37°C in a Victor-3 Plate Reader (Perkin Elmer) [34,35].

### In vitro peptide degradation assay

2nmol of >98% pure peptides (Bio-Synthesis, USA) were digested with 15μg of whole cell extracts, normalized to actin levels, at 37°C in 50μL of degradation buffer (50mM Tris-HCl, 137mM potassium acetate, 1mM MgCl_2_, and 1mM ATP, pH7.4, pH5.5, or pH4.0) [37,45]. At various time points the reaction was stopped with 2.5μL of 100% formic acid (FA) and peptide fragments were purified by 5% trichloroacetic acid precipitation.

### Mass spectrometry analysis

Peptides in the digestion mix were identified by in house mass spectrometry. Equal amounts of peptide degradation samples were injected into a Nano-HPLC (Eksigent) and online nanosprayed into an Orbitrap mass spectrometer (LTQ Orbitrap Discovery, Thermo) with a flow rate of 400nL/min. A Nano cHiPLC trap column (200μm x 0.5mm ChromXP c18-CL 5μm 120Å; Eksigent) was used to remove salts in the sample buffer. Peptides were separated in a Nano cHiPLC column (75μm x 15cm ChromXP c18-CL 5μm 300Å; Eksigent) over a gradient of 2% to 40% buffer B (buffer A: 0.1% FA in water; buffer B: 0.1% FA in acetonitrile) and mass spectra were recorded in the range of 370 to 2000Daltons. In tandem MS/MS mode, the eight most intense peaks were selected with a window of 1Da and fragmented. The collision gas was helium, and the collision voltage was 35V. Masses in the mass spectra were searched against source peptide databases with Proteome Discoverer (Thermo Scientific). The integrated area under a peptide peak is proportional to its abundance. Each sample was run on the mass spectrometer at least twice.

### Intracellular stability of optimal epitopes

One nmol of highly purified peptide was degraded in 15μg of whole cell extracts at 37°C in degradation buffer at pH7.4 or pH4.0 [37]. Aliquots were taken at 0, 10, 30, and 60 minutes, and the reaction was stopped with 2.5μL of 100% TFA. The remaining peptide at each time point was quantified by reversed-phase HPLC (RP-HPLC; Waters). 100% represents the amount of peptide detected at time point 0 calculated as the area under the peptide peak. A stability rate of each peptide was calculated by a nonlinear regression (one-phase exponential decay) of the degradation profile obtained over a 60-minute incubation [35,44]. Peptides incubated in buffer without cell extracts were used as controls.

### Statistical analysis

Spearman’s rank correlation coefficient was used to examine bivariate associations. The Kruskal-Wallis test was used to compare measurements between groups. In figures, p-value criteria are assigned as * p<0.05, ** p<0.01 and *** p<0.001. Statistical analyses were conducted using GraphPad Prism (GraphPad Prism Software, USA).

## Results

### Cross-presentation of the immunodominant HLA-B57-restricted Gag p24 TW10 and KF11 epitopes is more efficient than that of subdominant ISW9 epitope

**Fig 1:**
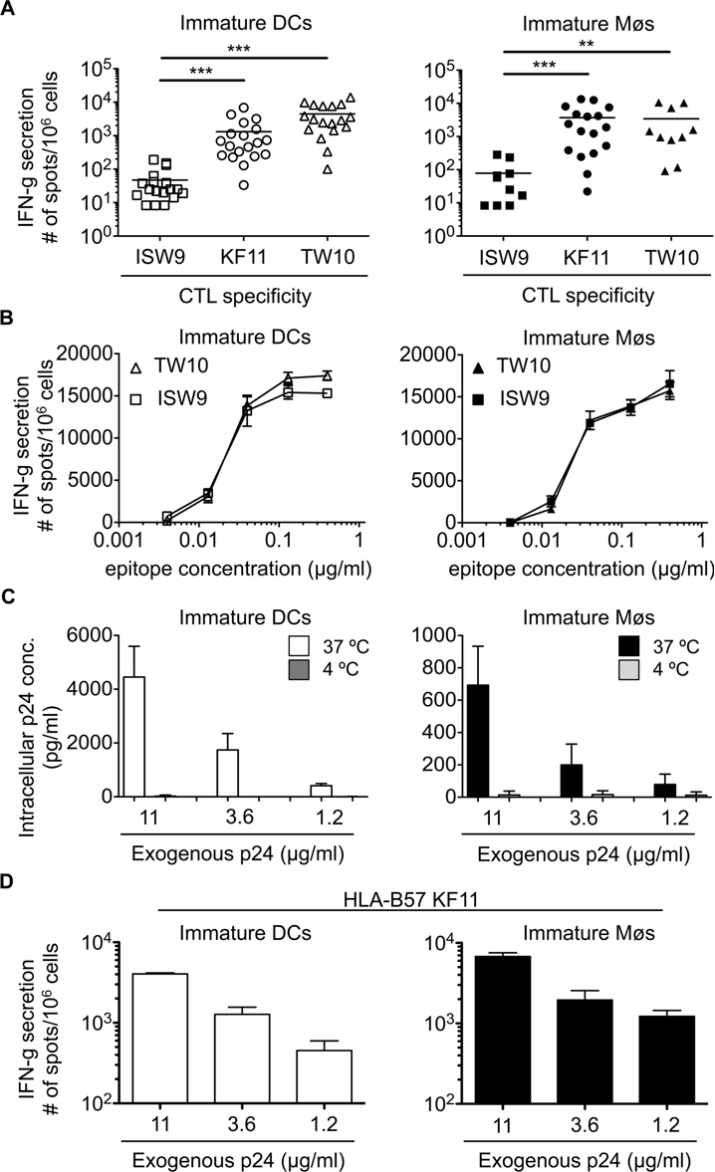
The immunodominant HLA-B57-restricted TW10 and KF11 epitopes are more efficiently cross-presented than subdominant ISW9 epitope. A. Immature DCs (open symbols) and Møs (solid symbols) were incubated with recombinant HIV-1 p24 protein and used as antigen presenting cells in an overnight IFN-gamma ELISPOT assay with HLA-B57 ISW9-specific (☐), KF11-specific (○), and TW10-specific (Δ) CTL clones as effector cells. CTL responses in form of spot forming cells are shown for n≥10 donors. B. CTL responses to immature DCs (open symbols) and Møs (solid symbols) pulsed with different concentrations of optimal epitope B57-ISW9 (☐) or B57-TW10 (Δ) were measured in an overnight IFN-gamma ELISPOT assay. Data are representative of two independent experiments with different donors. Due to the limited number of DCs and Møs generated from HLA-B57^+^ donors, the titration of epitopes was only done for B57-ISW9 and B57-TW10. C. Immature DCs (open bars) and immature Møs (solid bars) were pre-treated with a cocktail of protease inhibitors to reduce intracellular degradation before addition of different concentrations of recombinant HIV-1 p24 protein at 37°C or 4°C. Internalized p24 protein was determined by a standard p24 ELISA assay using whole cell extracts from lysed DCs and Møs. Results are from three independent experiments with different donors and show mean ± SD. D. Immature DCs (open bars) or immature Møs (solid bars) were incubated with different concentrations of recombinant HIV-1 p24 protein and used as antigen presenting cells to B57-KF11-specific CTLs. Responses in form of spot forming cells are shown. Data are representative of two independent experiments with different donors.

We analyzed the cross-presentation of the three optimally defined HLA-B57 restricted HIV epitopes originating from HIV-1 p24 protein by immature monocyte-derived DCs and Møs: subdominant B57-ISW9 (ISPRTLNAW, aa 15–23 in Gag p24), dominant B57-KF11 (KAFSPEVIPMF, aa 30–40 in Gag p24), and dominant B57-TW10 (TSTLQEQIGW, aa 108–117 in Gag p24) [46,47]. B57-ISW9-specific CTL responses to cross-presenting DCs were 28-fold and 94-fold lower compared with B57-KF11 and B57-TW10-specific responses, respectively (Fig. 1A, left panel). Similar results were observed with cross-presenting Møs, with 47-fold lower B57-ISW9-specific CTL responses compared with B57-KF11 and B57-TW10-specific CTL responses (Fig. 1A, right panel). DCs and Møs pulsed with increasing amounts of synthetic ISW9 or TW10 peptides similarly activated epitope-specific CTLs (Fig. 1B). Since a previous study showed comparable affinities of ISW9, KF11 and TW10 peptides for HLA-B57 [48], our results suggest that differences in CTL responses to cross-presenting DCs and Møs are not due to differences in peptide avidity among the clones, but likely to differential epitope production. To ensure that epitopes cross-presented by DCs and Møs were endogenously processed, we measured the intracellular concentrations of HIV-1 p24 protein after uptake at 37°C or 4°C. In both cell subsets the intracellular concentration of HIV p24 increased with the amount of p24 used for uptake at 37°C whereas the uptake at 4°C was minimal (Fig. 1C). Immature Møs showed at least 5-fold lower intracellular p24 concentrations than DCs, which may indicate a faster degradation of internalized protein by Møs [49]. Moreover, B57-KF11-specific CTL responses increased with the amount of exogenous p24 protein added to cells, in accordance with higher amount of intracellular p24 leading to higher amount of peptide presentation (Fig. 1D). Together, these data show that the higher CTL responses against dominant TW10 and KF11 epitopes after uptake of p24 by DCs and Møs are due to cross-presentation of higher amounts of both peptides compared with subdominant ISW9.

### Protease activities in cross-presentation competent cell compartments differently affect processing of HIV-1 epitopes in DCs and Møs

**Fig 2:**
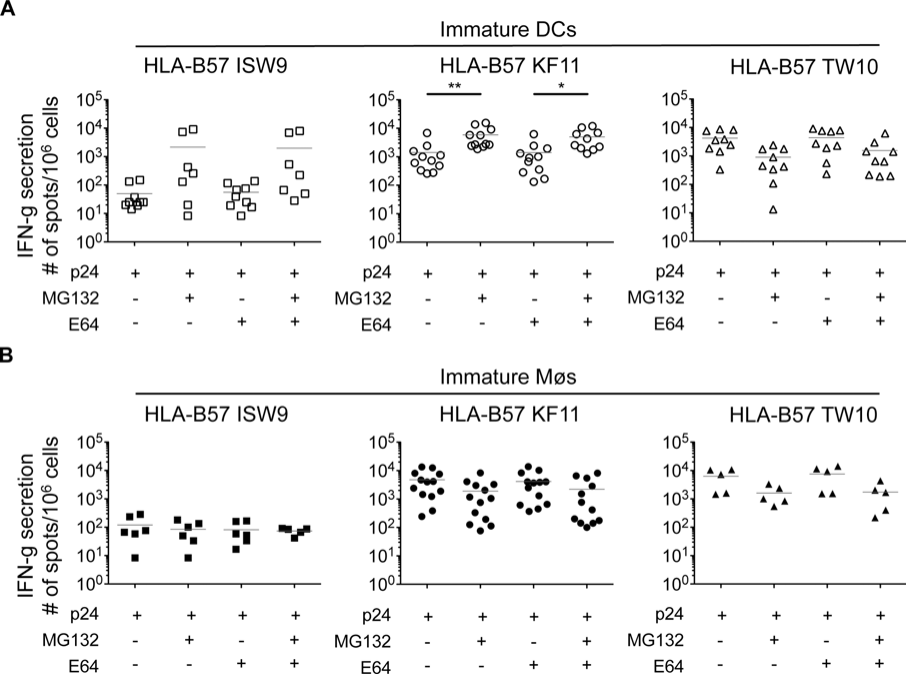
Inhibition of proteasomes increases B57-KF11 and B57-ISW9 epitope cross-presentation by DCs but not Møs (A) Immature DCs (open symbols) and (B) immature Møs (solid symbols) were pretreated with proteasome inhibitor (MG132) or a broad inhibitor of cysteine proteases (E64) before addition of recombinant HIV-1 p24 protein. CTL responses to HLA-B57 ISW9-specific (☐), KF11-specific (○), and TW10-specific (Δ) were measured as described in Fig. 1. Similarly, incubation of DCs with epoxomicin, a selective chymotrytpic proteasome inhibitor, increased HLA-B57-KF11-specific CTL responses by 11-fold. Results are from n≥5 donors.

We aimed to identify factors contributing to the production or destruction of the three epitopes in each cell type. Incubation of immature DCs with proteasome inhibitor MG132 resulted in a 43-fold and 4-fold increased presentation of B57-ISW9 and B57-KF11 epitopes, respectively, suggesting that proteasomal degradation of epitope-containing peptides limited the amount of ISW9 and KF11 available for presentation (Fig. 2A). In contrast, inhibition of cysteine proteases by E64 had no effect on the cross-presentation of both epitopes, indicating that fragments escape early into the cytosol before trafficking to compartments with high cysteine protease activity. B57-TW10-specific CTL responses to cross-presenting immature DCs decreased approximately 3-fold upon inhibition of proteasomes, suggesting that proteasomal processing is required for efficient presentation of TW10. In contrast to DCs, the cross-presentation of B57-ISW9 by immature Møs was not affected upon inhibition of proteasomes, suggesting that the cross-presentation of ISW9 in Møs is proteasome-independent (Fig. 2B). B57-KF11 and B57-TW10 CTL responses decreased 2- and 3-fold respectively upon proteasome inhibition with MG132 or epoxomicin in Møs, suggesting that the processing of both epitopes requires proteasome processing in Møs. Inhibition of cysteine proteases in Møs did not affect the cross-presentation of ISW9, KF11 and TW10. Together, these results indicate that cross-presentation of HIV-1 p24 involves distinct proteases in DCs and Møs, which can be essential or detrimental for the processing of epitopes.

### The immunodominant epitopes B57-KF11 and B57-TW10 are more efficiently produced in cytosolic and endolysosomal extracts than subdominant epitope B57-ISW9

Exogenous antigens internalized by DCs and Møs first encounter several proteases in endo- and lysosomes [27], before presentation or additional degradation in the cytosol. In line with previous studies we observed lower omnicathepsin and cathepsin S activities in DCs compared with Møs [49], which further decreased upon maturation of DCs as shown by an inverse correlation between both activities and the % of mature DCs (S1A Fig). To assess how degradation of HIV peptides along the cross-presentation pathway of immature and TLR ligand-stimulated DCs and Møs may contribute to shaping immunodominance patterns, we used a previously developed degradation assay recapitulating degradation in the cross-presentation compartments [37]. This assay allows the simultaneous analysis of degradation products by cytosolic, endosomal and lysosomal peptidases from the same cells using mass spectrometry. Omnicathepsin and cathepsin S activities measured in live intact cells correlated to their matching cell extracts, as previously demonstrated for cytosolic proteases [35] (S1B Fig) and could be activated at different pH values, in accordance with differential cathepsin activation in endosomes and lysosomes (S1C Fig) [37].

**Fig 3:**
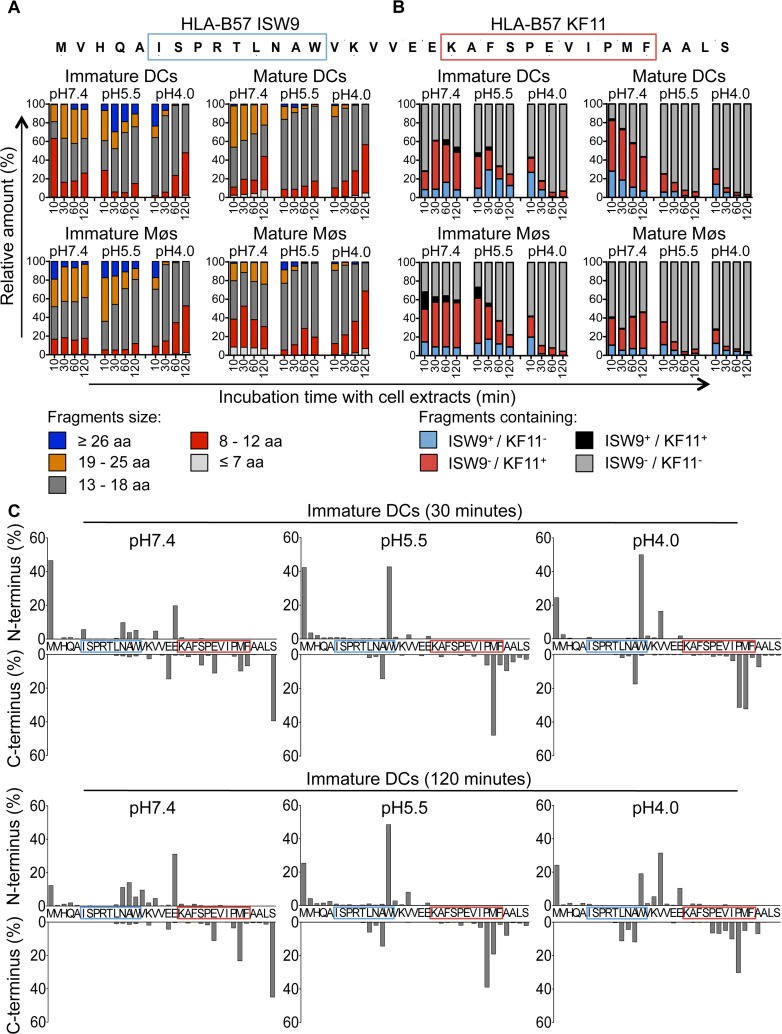
The immunodominant epitope B57-KF11 is more efficiently produced in cross-presentation-competent compartments than subdominant epitope B57-ISW9. A. Two nmol of p24–35mer (MVHQAISPRTLNAWVKVVEEKAFSPEVIPMFAALS, aa 10–44 in Gag p24) were degraded in 15μg of whole cell extracts from immature and mature DCs and Møs for 10, 30, 60, and 120 minutes in degradation buffer at pH7.4, pH5.5 or pH4.0. Degradation products identified by mass spectrometry were grouped according to their lengths of fragments: equal or longer than 26 aa (blue), 19–25 aa (orange), 13–18 aa (gray), 8–12 aa (red), and fragments equal or shorter than 7 aa (light gray). The peak area of each identified peptide was calculated with Proteome Discoverer and the contribution of each category of peptides to the total intensity of all degradation products is shown at each time point. B. All degradation products of p24–35mer were identified as described in (A). Peptides were grouped into fragments containing B57-ISW9 and B57-KF11 epitopes (black), containing only B57-KF11 epitope (red), containing only B57-ISW9 epitope (blue), or neither epitope (gray), respectively. C. Cleavage patterns of p24–35mer incubated with whole cell extracts from immature DCs for 30 minutes (upper panel) or 120 minutes (lower panel) at pH7.4, pH5.5, and pH4.0 are shown as the contribution of each cleavage site, presented as cleavage N-terminal or C-terminal to a specific amino acid, to the total intensity of all degradation products. For (A-C) data are representative of three independent experiments with three different donors.

Degradation of a synthetic 35-mer peptide containing the epitopes B57-ISW9 and B57-KF11 (MVHQAISPRTLNAWVKVVEEKAFSPEVIPMFAALS, aa 10–44 in Gag p24) [34,35,37] showed the production of peptides of variable lengths at different pH values over time (S2 Fig). To assess and compare the production of peptides in each cell subset and cell compartment, we used the area under each peptide peak identified by mass spectrometry, which we previously showed to be proportional to the amount of the corresponding peptide [35,50]. Peptides were grouped according to their lengths or epitope content, and the contribution of each category of peptides to the total degradation products was calculated for each time point. Degradation at pH4.0 in cell extracts from immature DCs yielded shorter fragments compared with degradation at pH7.4, with majority of fragments being 8–12 and 13–18 aa long and contributing to 45% and 51% of total peptide intensity at 120 minutes, respectively (Fig. 3A, upper left panel). The degradation of fragments containing both epitopes (ISW9^+^/KF11^+^) resulted in the preferential production of B57-KF11 epitope-containing fragments (ISW9^-^/KF11^+^), and only small amounts of B57-ISW9 epitope-containing fragments (ISW9^+^/KF11^-^) in extracts of immature DCs at all pH values tested (Fig. 3B, upper left panel). KF11- and ISW9-containing fragments were produced more efficiently at pH7.4 than at pH5.5 and pH4.0, indicating a higher presentation in the direct presentation pathway, or if epitope precursors escape from endolysosomes. Similar results were observed for immature Møs (Fig. 3A/B, lower left panel), in line with comparable cytosolic and endocytic hydrolytic activities in immature DCs and Møs [35]. Degradation of the 35-mer in extracts from DCs and Møs matured with LPS yielded similar degradation patterns with fragments of comparable lengths and higher amounts of fragments containing immunodominant epitope B57-KF11 (Fig. 3A/B, right panel).

We further analyzed the cleavage patterns by measuring the relative amount of fragments with a specific N terminus or C terminus (Fig. 3C, upper or lower graph of each panel). After 30 minutes of degradation at pH7.4 several minor cleavage sites produced ISW9- and KF11-containing fragments, whereas at pH5.5 and pH4.0 the generation of fragments with a Tryptophan at the N terminus destroyed ISW9 and fragments with a Methionine and Proline at the C terminus destroyed KF11. Further trimming resulted in the appearance of new N- and C-terminal cleavage sites, which still preserved KF11-containing peptides at pH7.4, whereas at pH5.5 and pH4.0 both epitopes were further destroyed. These data indicate that this p24 35-mer is sensitive to degradation in all three cell compartments in DCs and Møs, and favors the production of dominant epitope KF11 over that of subdominant ISW9, in line with the more efficient cross-presentation of KF11 and the rescue of ISW9 in the presence of protease inhibitors.

Moreover, we analyzed the production of 16 well described HIV CD8^+^ and CD4^+^ T cell epitopes [46] and epitope precursors, defined as N-terminal extended epitopes, located in this 35-mer (S3A–S3B Fig). Peptides were produced in extracts of both cell subsets at all pH values tested (B57-KF11, B15-HL9, A25-QW11, B57-FF9, B57-KP9), preferentially produced at pH7.4 (B57-ISW9, A02-TV9), or at pH5.5 and pH4.0 (B45-VI11, B15-VF9, B44-EV9) or not produced at any time (B07-SV9). These results highlight a variable production or degradation of epitopes in different cell compartments, which may affect their capacity to activate CD8^+^ or CD4^+^ T cells during infection or cross-presentation.

**Fig 4:**
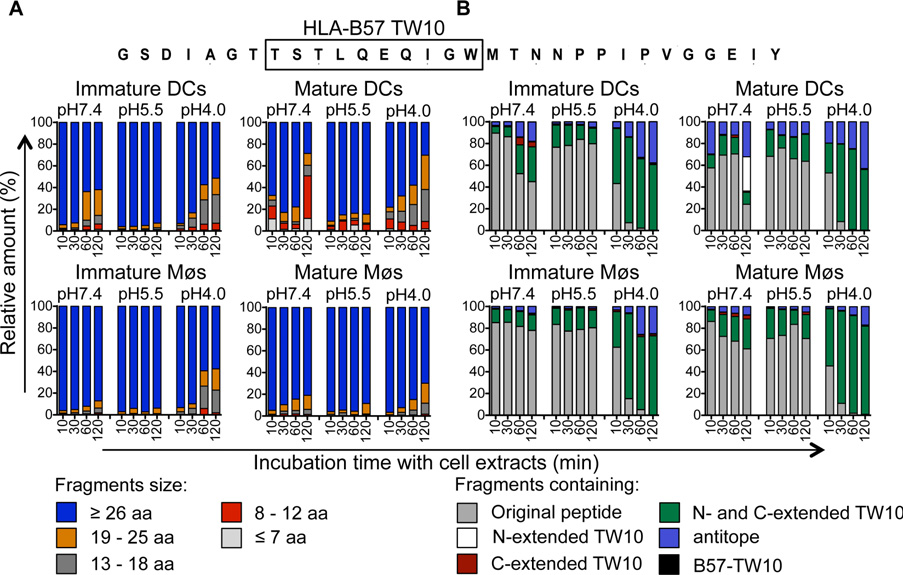
Slow degradation of TW10-containing peptides results in high amounts of B57-TW10 available for cross-presentation. A. Two nmol of p24–31mer (GSDIAGTTSTLQEQIGWMTNNPPIPVGGEIY, aa 101–131 in Gag p24) were degraded in 15μg of whole cell extracts from immature and mature DCs and Møs for 10, 30, 60, and 120 minutes in degradation buffer at pH7.4, pH5.5 or pH4.0. Degradation products identified by mass spectrometry were grouped according to their lengths of fragments as described in Fig. 3. The contribution of each category of peptides to the total intensity of all degradation products is shown at each time point. B. All degradation products of p24–31mer were grouped into fragments containing B57-TW10 (black), B57-TW10 epitope with N-terminal extensions (white), B57-TW10 epitope with C-terminal extensions (red), B57-TW10 epitope with N- and C-terminal extensions (green), antitopes defined as fragments lacking B57-TW10 (blue), or the original peptide (gray), respectively. The contribution of each category of peptides to the total intensity of all degradation products is shown at each time point.

We next extended the analysis to another HIV-1 Gag p24-derived peptide containing the epitope B57-TW10 (GSDIAGTTSTLQEQIGWMTNNPPIPVGGEIY, aa 101–131 in Gag p24), dominant in HIV acute infection (Fig. 4). In contrast to p24 35-mer, the majority of degradation products identified after 10 to 120 minutes in extracts from immature DCs and Møs at pH7.4, pH5.5 and pH4.0 represented the original fragment or long fragments of mostly >26aa (Fig. 4A), containing the TW10 epitope with N- and C-terminal extensions (Fig. 4B). Similarly, degradation in extracts from mature DCs and Møs showed comparable kinetics of degradation and resulted in the production of fragments with similar lengths. Accordingly the few cleavage sites identified after 30 or 120 minutes were mostly located outside B57-TW10 at all three pH values, protecting the antigenic peptide from degradation, in line with the highly efficient cross-presentation of B57-TW10 (S4 Fig).

The relative resistance of the B57-TW10-containing fragment to intracellular degradation contrasted with the rapid degradation of the p24 fragment containing B57-ISW9 and B57-KF11 which may contribute to a higher amount of TW10-containing peptide available for presentation by direct or cross-presentation, thus contributing to the dominance of TW10-specific CTL responses during acute HIV infection.

### Limited degradation of RK9-containing fragments results in efficient cross-presentation of the immunodominant HLA-A03 RK9 epitope by DCs and Møs

**Fig 5:**
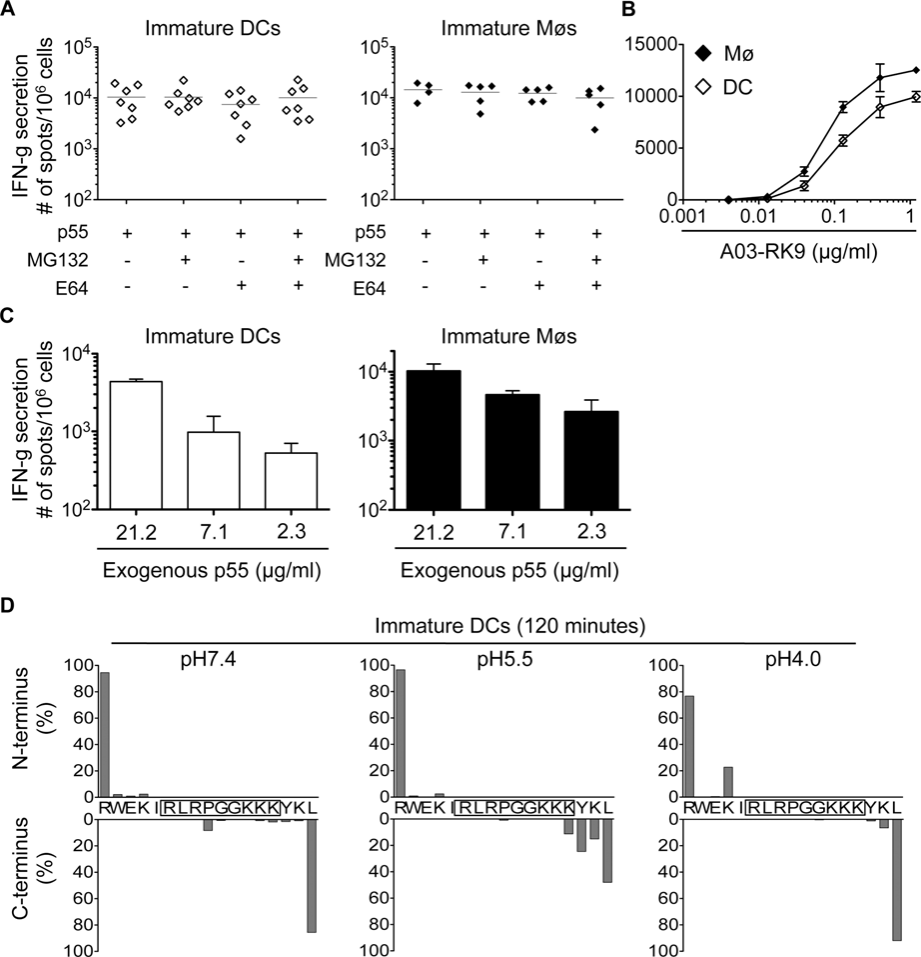
Limited degradation of RK9-containing fragments results in cross-presentation of high amounts of HLA-A03 RK9 epitope. A. Immature DCs (◇) or Møs (◆) were incubated with recombinant HIV-1 p55 protein and used as antigen presenting cells in an overnight IFN-gamma ELISPOT assay with HLA-A03 RK9-specific CTLs as effector cells. CTL responses in form of spot forming cells are shown for n≥5 donors. B. CTL responses to immature DCs (◇) and immature Møs (◆) pulsed with different concentrations of optimal epitope A03-RK9 were measured as described before. C. Immature DCs (open bars) or Møs (solid bars) were incubated with different concentrations of recombinant HIV-1 p55 protein and used as antigen presenting cells in an overnight IFN-gamma ELISPOT assay with HLA-A03 RK9-specific CTLs as effector cells. CTL responses in form of spot forming cells are shown. D. Cleavage patterns of p17–17mer incubated with whole cell extracts from immature DCs for 120 minutes at pH7.4, pH5.5, and pH4.0 are shown as the contribution of each cleavage site, presented as cleavage N-terminal or C-terminal to a specific amino acid, to the total intensity of all degradation products. For (B-D) data are representative of two independent experiments with different donors.

We next analyzed the cross-presentation of another immunodominant epitope located in a different HIV-1 protein and restricted by a different HLA allele. The A03-RK9 epitope (RLRPGGKKK, aa 20–28 in Gag p17) is efficiently produced in the endogenous processing pathway for presentation to A03-RK9-specific CTLs [17,44]. Immature DCs and Møs, incubated with recombinant HIV-1 p55 protein elicited A03-RK9-specific CTL responses as strong as cells exogenously pulsed with 1.2 ug/ml RK9 (Fig. 5A-B). RK9 cross-presentation was not affected by inhibition of proteasome or cysteine proteases, in line with the limited sensitivity of proteasome and cathepsin-mediated degradation resulting in high amounts of peptide for maximum T cell stimulation (Fig. 5A). The incubation of DCs or Møs with different concentrations of p55 protein resulted in concentration-dependent A03-RK9-specific CTL responses (Fig. 5C). In vitro degradation of HIV-1 p17-derived peptide containing the A03-RK9 epitope (RWEKIRLRPGGKKKYKL, aa 15–31 in p17) showed minimal degradation of A03-RK9 at all pH values tested (Fig. 5D). These data indicate that the limited degradation of this peptide may result in more fragments available for cross-presentation compared with B57-ISW9 and B57-KF11 epitopes, thus contributing to immunodominance of RK9-specific CTL responses.

### Intracellular epitope stability of HIV-1 epitopes in cytosolic and lysosomal cell extracts follows CTL responses hierarchy

**Fig 6:**
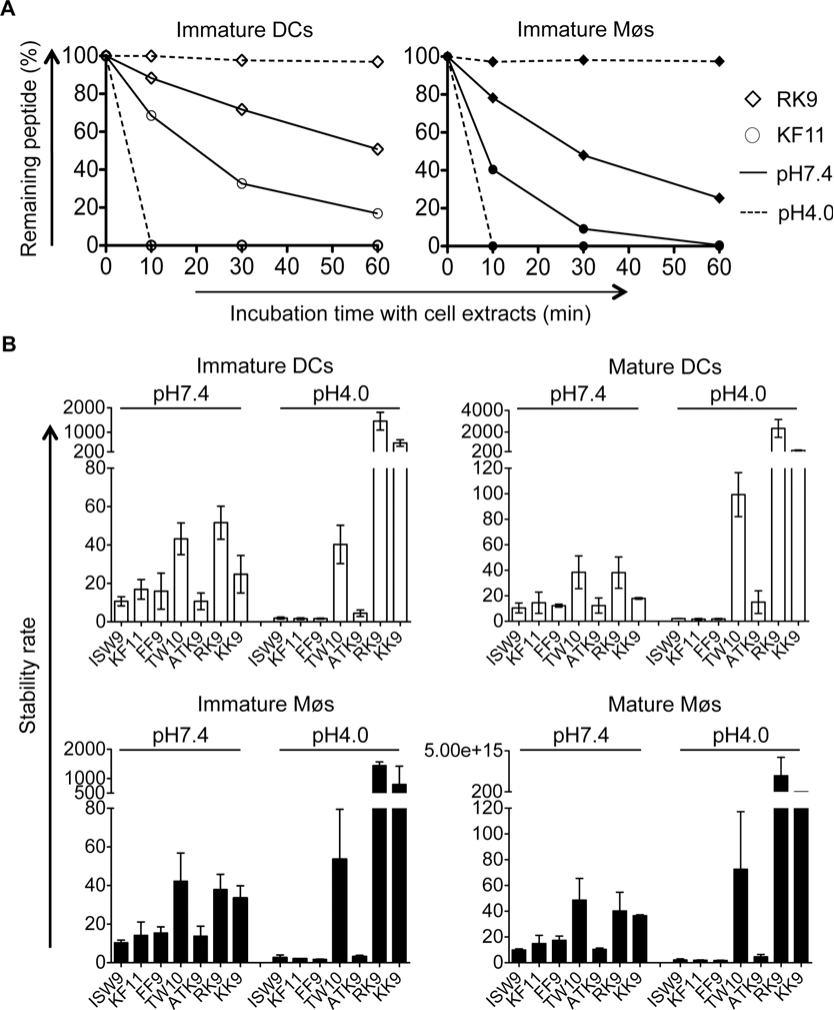
The intracellular stability of HIV-1 epitopes in the cytosol and lysosomes follows CTL responses hierarchy. A. One nmol of highly purified HLA-B57-restricted KF11 (○) and HLA-A03-restricted RK9 (◇) were degraded in 15μg of immature DC or Mø extracts (open or solid symbols, respectively) in degradation buffer at pH7.4 (solid line) or pH4.0 (dashed line). Degradation products were analyzed by RP-HPLC after 10, 30, and 60 minutes. 100% represents the amount of peptide detected at time 0, calculated as the surface area under the peptide peak. B. The stability rate of optimal epitopes B57-ISW9, B57-KF11, B57-FF9, B57-TW10, A11-ATK9, A03-RK9, and A03-KK9 at pH7.4 and pH4.0 was calculated by a nonlinear regression (one-phase exponential decay) of the degradation profile obtained over a 60-minute incubation in extracts of immature and mature DCs (upper panel) and Møs (lower panel). Bars represent the mean ± SD of three independent experiments for each epitope with extracts from different healthy donors.

The variable stability of epitopes in cytosolic extracts of PBMCs contributes to the amount of peptide available for presentation to T cells [44,50,51]. We hypothesize that peptide stability in cytosol and endolysosomes of DCs and Møs may contribute to the relative efficiency of cross-presentation of immunodominant epitopes. We first measured the stability of A03-RK9, a dominant epitope in the acute phase, and B57-KF11, a dominant epitope in the chronic phase, in whole cell extracts of immature DCs and Møs at pH7.4 and pH4.0. In cell extracts of immature DCs, the B57-KF11 epitope was 10-fold faster degraded at pH4.0 than at pH7.4 (half-lives of 1.9 minutes versus 20 minutes) whereas the A03-RK9 epitope was 20-fold faster degraded at pH7.4 than at pH4.0 (half-lives of 61 minutes versus 1223 minutes) (Fig. 6A, left panel). Similar results were observed in cell extracts from immature Møs (Fig. 6A, right panel). We next compared the intracellular stability of seven well-defined dominant and subdominant MHC I epitopes located in HIV Gag p24, Gag p17 and RT, and examined whether their stability corresponded to immunodominance patterns observed in HIV infection. To rank epitopes we calculated a stability rate as done before [44,50]. In cell extracts of immature DCs at pH7.4 the dominant epitopes A03-RK9 and B57-TW10 showed approximately 5-fold and 4-fold higher stability rates compared with the subdominant epitopes B57-ISW9 and A11-ATK9 (AIFQSSMTK, aa 158–166 in RT), respectively (Fig. 6B, upper left panel). At pH4.0 we detected dramatically reduced stability rates for B57-ISW9, B57-KF11, B57-FF9, and A11-ATK9, indicating a more rapid proteolysis by proteases located in endo- and lysosomes. However, the observed stability rates of all epitopes formed the same hierarchy as seen at pH7.4. Similar results were observed in cell extracts of immature Møs (Fig. 6B, lower left panel). Moreover, the subdominant A03-KK9 epitope overlapping with the dominant A03-RK9 epitope had a 3-fold lower stability rate in both cell subsets at pH4.0, whereas the dominant and overlapping epitopes B57-KF11 (KAFSPEVIPMF, aa 30–40 in Gag p24) and B57-FF9 (FSPEVIPMF, aa 32–40 in Gag p24) [52] had comparable stability rates. In line with our previous study [35], the cytosolic stability rate of all epitopes was not affected upon maturation of DCs and Møs with LPS (Fig. 6B, upper and lower right panel). Together, these results show that the intracellular stability of optimal HIV epitopes is highly variable in DCs and Møs and in different cell compartments, but follows similar hierarchies and may contribute to differences seen in cross-presentation and immunodominance patterns observed in HIV infection.

### Frequent escape mutations in an immunodominant epitope reduce epitope production and peptide stability in the cross-presentation pathway

**Fig 7:**
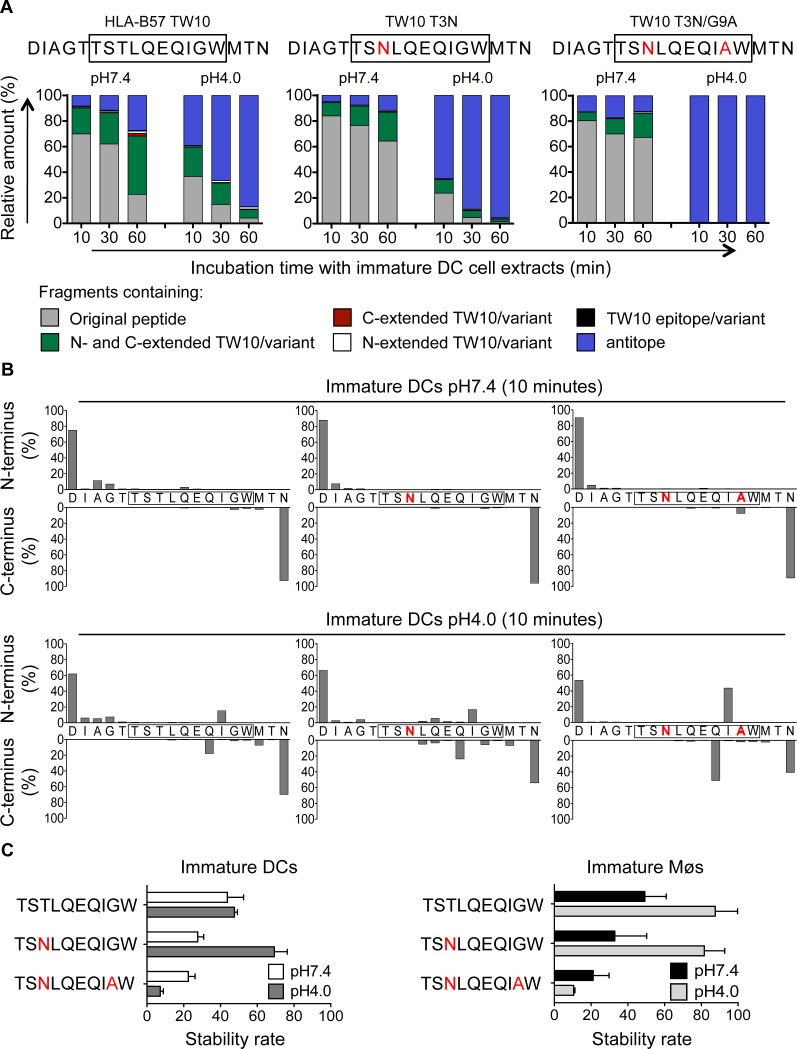
Escape mutations in immunodominant epitopes reduce epitope production and stability in the cross-presentation-competent cell compartments. A. Two nmol of 5-TW10–3 (DIAGTTSTLQEQIGWMTN, aa 103–120 in Gag p24), 5-TW10–3 T3N (DIAGTTSNLQEQIGWMTN), and 5-TW10–3 T3N/G9A (DIAGTTSNLQEQIAWMTN) were degraded in 15μg of whole cell extracts from immature DCs for 10, 30 or 60 minutes in degradation buffer at pH7.4 and pH4.0. Degradation products identified by mass spectrometry were grouped into fragments containing the optimal epitope TW10 or its mutant (black), the epitope/mutant with N-terminal extensions (white), the epitope/mutant with C-terminal extensions (red), the epitope/mutant with N- and C-terminal extensions (green), antitopes defined as fragments lacking part of the epitope/mutant (blue), or the original peptide (gray), respectively. The contribution of each category of peptides to the total intensity of all degradation products is shown at each time point. B. Cleavage patterns of 5-TW10–3, 5-TW10–3 T3N, and 5-TW10–3 T3N/G9A incubated with whole cell extracts from immature DCs for 10 minutes at pH7.4 (upper panel) or at pH4.0 (lower panel) are shown as the contribution of each cleavage site, presented as cleavage N-terminal or C-terminal to a specific amino acid, to the total intensity of all degradation products. For (A-B) data are representative of two independent experiments with different donors. C. One nmol of highly purified HLA-B57-restricted TW10, TW10-T3N or TW10-T3N/G9A mutants were degraded in 15μg of immature DCs or Møs extracts (right and left panel) in degradation buffer at pH7.4 or pH4.0. A stability rate was calculated as described before. Bars represent the mean ± SD of three independent experiments for each epitope with extracts from different healthy donors.

Immune pressure exerted by T cell immune responses leads to predictable mutations within and outside epitopes altering viral fitness, epitope processing and presentation [44]. In HLA-B57^+^ patients the TW10 epitope rapidly mutates at residues 3 and 9 during acute infection [53,54], and the dominant TW10 CTL response wanes while KF11-specific CTL responses become dominant [2,55]. To assess the impact of escape mutations on degradation patterns in the cross-presentation pathway, peptides containing the TW10 epitope or its naturally occurring variants TW10 T3N or TW10 T3N/G9A were degraded in whole cell extracts from immature DCs and Møs at pH7.4 and pH4.0 for 10, 30 and 60 minutes. Degradation of the two variants at pH7.4 showed comparable kinetics of disappearance of the original peptides (64–67% of original peptides left), whereas the WT showed faster degradation with 23% of the original peptide left after 60 minutes (Fig. 7A). However, both mutants generated less N- and C-extended TW10-containing peptides than the WT at pH7.4 (23% TW10 T3N variant, 19% TW10 T3N/G9A variant vs. 45% TW10 WT at 60 minutes). Degradation at pH4.0 demonstrated a very fast kinetic of degradation of the T3N/G9A peptide, and generation of a majority of fragments lacking part of the epitope (antitopes) (39% TW10 WT, 65% T3N variant, and 99% T3N/G9A variant of antitopes produced after 10 minutes). The analysis of the N- and C-terminal cleavage sites showed that TW10 WT and TW10 T3N sequences were spared from degradation at pH7.4, whereas a cleavage site between Tryptophane and Alanine partly destroyed the TW10 T3N/G9A variant within 10 minutes of degradation (Fig. 7B, upper panel). At pH4.0, in line with the faster degradation of the long peptides into antitopes, two major cleavage sites produced short antitopes with an Isoleucine at the N-terminus or a Glutamine at the C-terminus that partly destroyed TW10 WT, and more extensively TW10 T3N/G9A. These cleavage sites were 2.8-fold and 2.6-fold more pronounced in the TW10 T3N/G9A variant compared with the TW10 WT and the TW10 T3N variant (Fig. 7B, lower panel). We compared the cytosolic and lysosomal stability of TW10 epitope and its variants in DCs and Møs (Fig. 7C). Similar intracellular stabilities were observed for B57-TW10 and TW10 T3N in each compartment whereas the stability of TW10 T3N/G9A was reduced by 7- to 8-fold in both compartments. These results demonstrate that the low intracellular stability of TW10 T3N/G9A variant contribute to reducing the epitope presentation in both direct and cross-presentation pathways, in line with our previous findings in cells infected with virus containing TW10 WT or variants [44]. This represents the first demonstration of an escape mutation affecting the cross-presentation of an HIV epitope.

## Discussion

This study shows how degradation patterns in the cross-presentation pathway of APCs favor the production of immunodominant HIV epitopes and provides the first demonstration of antigen processing mutations affecting the cross-presentation of HIV epitopes by APCs to CTLs.

The production and presentation of HIV epitopes are affected by both the cell subsets and the cellular compartments in which antigen traffics [34–36,38]. Differences in the processing of epitopes between monocyte-derived DCs and Møs include the involvement of distinct sets of peptidases in the production or destruction of epitopes, and in the trafficking of degradation products. While proteasomes were involved in the degradation of B57-ISW9-containing fragments in DCs, its processing in Møs was proteasome-independent, suggesting possible differences in the trafficking of epitope-containing fragments and the involvement of proteases located in different subcellular compartments [33,56–58]. The degradation of peptides by specific proteases and the translocation across different cell compartments has been demonstrated to depend on the size of fragments [59,60], which may contribute to the observed differences in cross-presentation of B57-ISW9 and B57-KF11 by DCs and Møs upon proteasome inhibition. The formation of specific peptide pools [61] or DC-specific antigen storage compartments [62] may permit the fast cross-presentation of HIV peptides by DCs and Møs but this remains to be demonstrated. In addition, cell-specific peptidases such as a serine protease uniquely expressed in monocytes [63], may lead to cell-specific processing of epitopes and may contribute to the differential priming of immune responses by different tissue DC subsets observed in vivo [64–66]. However, further transcriptomics, proteomics [67] and functional analyses are needed to identify additional cell subset-specific peptidases that may shape epitope presentation by various APC subsets.

A major difference between DCs and Møs contributing to the ability of DCs for cross-priming is their capacity to tightly control endolysosomal pH at higher values than in Møs [68,69], which leads to lower cathepsin activities and slower degradation rates of proteins [49]. However lower peptidase activities are not always corresponding to better epitope production, as we identified epitopes processed in higher amount in Møs or in monocytes than in DCs [35]. Although technically still challenging it will be essential to determine the relative amount of peptides required for priming or activation of T cell responses by DCs or other infectable cell subsets, and how this will affect the capacity of T cells to recognize infected targets and clear infection.

The level of peptidase activities in a given cell type and compartment, and the sensitivity of a given antigenic sequence to degradation in this compartment shapes the amount of epitope available for presentation [70]. In the cytosol the degradation profiles of proteins into epitopes [17,42] and the intrinsic stability of HIV peptides before loading onto MHC [35,44,51] determine the timing and amount of peptide available for presentation and are defined by specific motifs. It is likely that these steps in the endolysosomal pathway will be driven by motifs that still remain to be identified.

A direct consequence of this sequence- and compartment-dependent degradation of proteins is the impact of HLA-restricted mutations on HIV epitope processing and cross-presentation. Immune pressure selects variants impairing viral fitness and/or epitope presentation, reducing binding to MHC or to the TCR [71]. Flanking mutations have been shown to prevent the processing of epitopes [16,40,42,72] and intraepitopic mutations can destroy epitopes [44,73,74], which demonstrates the adaptation of HIV to antigen processing in the cytosol. This study provides the first demonstration that a frequently detected HLA-restricted mutation during acute infection affects the cross- and direct presentation of an epitope in DCs and Møs. While the WT epitope B57-TW10, dominant in the acute phase, is efficiently processed and highly stable in endo-lysosomes and the cytosol of DCs and Møs its mutant is degraded faster than WT. The lower amount of epitope available for presentation may lead to less epitope presented to T cells and therefore results most likely in subdominant responses, which coincides with a shift in immunodominance toward B57-KF11 in HLA-B57 persons during chronic HIV infection [2,55].

A vaccine eliciting the same immunodominance patterns as natural infection cannot be successful at preventing or clearing HIV infection. Breaking natural immunodominance and targeting immune responses towards protective epitopes [55] is required in the design of a T cell arm of vaccine strategies [75,76]. The combination of sequence alterations to modulate epitope production and intracellular peptide stability and to break natural immunodominance, the use of adjuvants modulating epitope processing as well as the targeting of immunogens to specific cell compartments offers ways to modulate epitope presentation to induce protective immunity in HIV infection and beyond.

## Supporting Information

S1 Fig Lysosomal activities in DC and Mø cell extracts reflect activities in live cells. A. Omnicathepsin and cathepsin S activities in immature and TLR-matured DCs were plotted against the percentage of CD86+ CD83+ DCs for each experiment. Surface expression was analyzed by flow cytometry. Comparison by Spearman test is indicated. n≥26 measurements. B. Omnicathepsin and cathepsin S hydrolytic activities measured in live intact immature or mature DCs (◇) and Møs (◆) were plotted against their activities in corresponding cell extracts at pH4.0. A partial correlation on Spearman ranked data was performed to control for cell type-dependent effects. n≥30 measurements. C. Cathepsin D, cathepsin S, cathepsin B, and omni cathepsin activities (combined cathepsin S, L, B activities) were measured with specific fluorogenic substrates in whole cell extracts of immature DCs at pH4.0, pH5.5, and pH7.4, respectively. Mean ± SD is shown for n≥5 independent donors.
(TIF)

S2 Fig Degradation of a HIV-1 p24 35mer in DC cell extracts at pH4.0, pH5.5 and pH7.4. Peptides containing the epitopes B57-ISW9 and B57-KF11 (black bars), B57-ISW9 epitope (blue bars), B57-KF11 epitope (red bars) or lacking both epitopes (gray bars) were identified by mass spectrometry. Optimal B57-ISW9 (blue star) and B57-KF11 (red star) are indicated. Data represent one of three independent experiments from different donors.
(TIF)

S3 Fig Variable production of 16 HIV-1 epitopes in cytosolic and endo-lysosomal extracts of DCs and Møs. A. The map shows the location of 12 MHC-I epitopes (black arrows) and 4 MHC-II epitopes (gray arrows) within the sequence of Gag p24–35mer (aa 10–44). B. Summary of the relative amount of optimal epitopes and corresponding N-terminal extensions detected by mass spectrometry after 10, 30, 60, and 120 minutes degradation in extracts of immature DCs, mature DCs, immature Møs, mature Møs at pH7.4, pH5.5 and pH4.0. Epitope precursors, defined as peptides with the correct C-terminus and extended by up to three residues at the N-terminus, could be further trimmed in the ER. Numbers represent contribution of optimals and N-extended optimals to the total intensity of all degradation products at each time point. The presence of optimal epitopes is indicated (*). Data represent one of three mass spectrometry analyses from independent experiments.
(TIF)

S4 Fig Limited degradation of TW10-containing fragments in cross-presentation-competent compartments of immature DCs. Cleavage patterns of p24–31mer (aa 101–131 in Gag p24) incubated with whole cell extracts from immature DCs for 30 minutes (left panel) or 120 minutes (right panel) at pH7.4, pH5.5, and pH4.0 are shown as the contribution of each cleavage site, presented as cleavage N-terminal or C-terminal to a specific amino acid, to the total intensity of all degradation products. Data are representative of three independent experiments with three different donors.
(TIF)
