# Supplementary figures and images for: Variable Processing and Cross-presentation of HIV by Dendritic Cells and Macrophages Shapes CTL Immunodominance and Immune Escape
Source: PLoS Pathog. 2015 Mar 17;11(3):e1004725. doi: 10.1371/journal.ppat.1004725 (PMC4364612; doi:10.1371/journal.ppat.1004725)

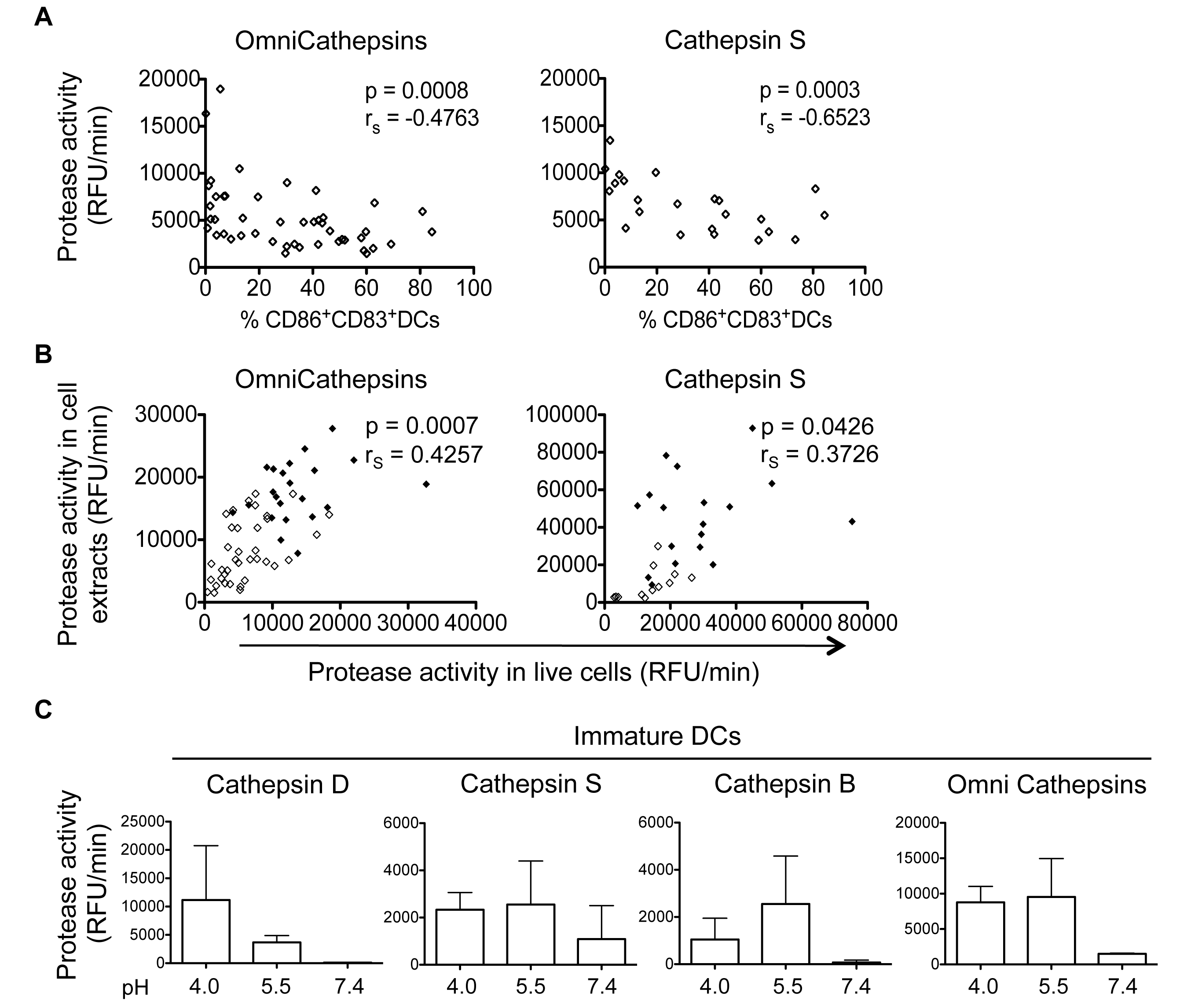

Supplement: S1 Fig — A. Omnicathepsin and cathepsin S activities in immature and TLR-matured DCs were plotted against the percentage of CD86+ CD83+ DCs for each experiment. Surface expression was analyzed by flow cytometry. Comparison by Spearman test is indicated. n≥26 measurements. B. Omnicathepsin and cathepsin S hydrolytic activities measured in live intact immature or mature DCs (◇) and Møs (◆) were plotted against their activities in corresponding cell extracts at pH4.0. A partial correlation on Spearman ranked data was performed to control for cell type-dependent effects. n≥30 measurements. C. Cathepsin D, cathepsin S, cathepsin B, and omni cathepsin activities (combined cathepsin S, L, B activities) were measured with specific fluorogenic substrates in whole cell extracts of immature DCs at pH4.0, pH5.5, and pH7.4, respectively. Mean ± SD is shown for n≥5 independent donors. (TIF) [file ppat.1004725.s001.tif]

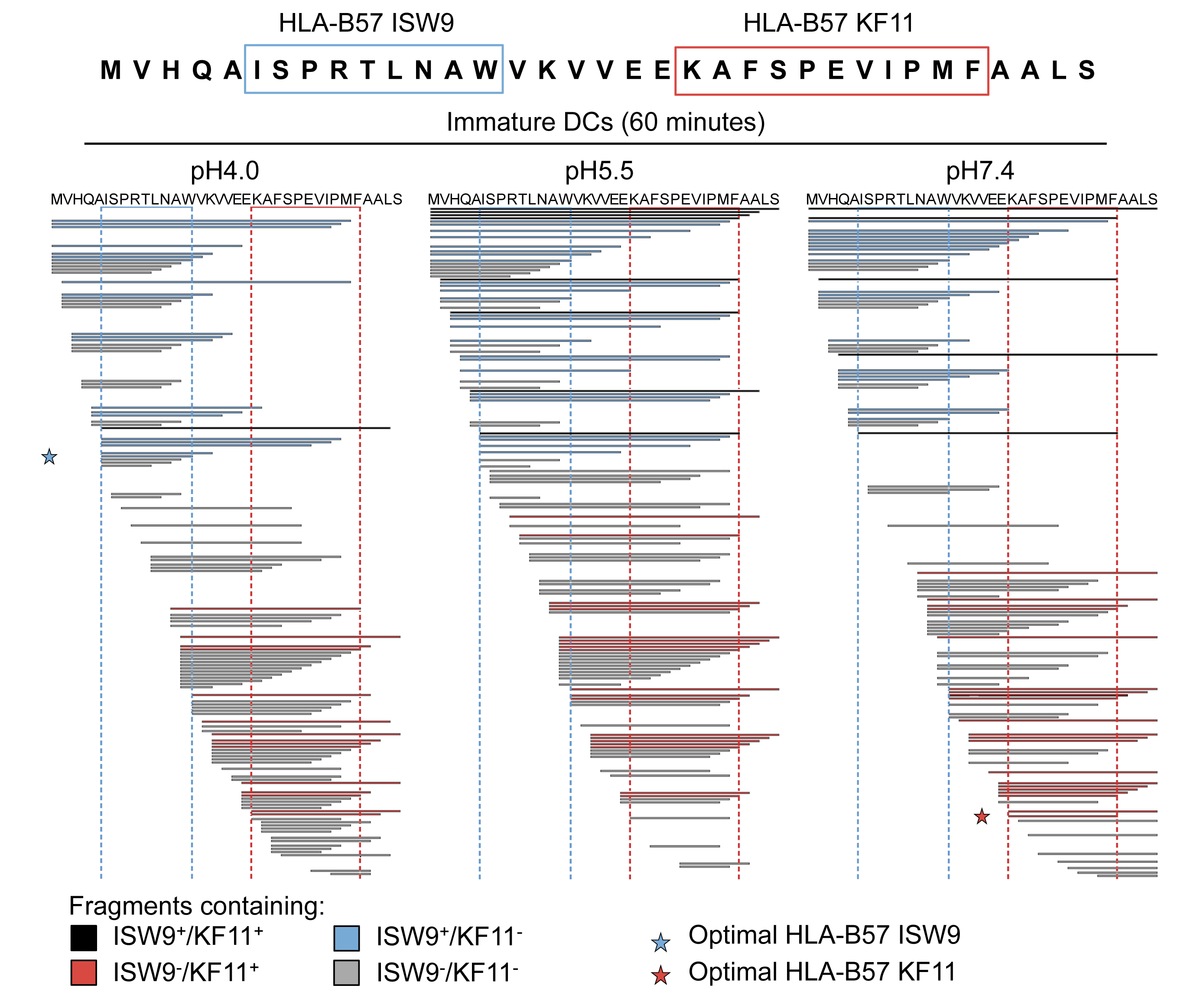

Supplement: S2 Fig — Peptides containing the epitopes B57-ISW9 and B57-KF11 (black bars), B57-ISW9 epitope (blue bars), B57-KF11 epitope (red bars) or lacking both epitopes (gray bars) were identified by mass spectrometry. Optimal B57-ISW9 (blue star) and B57-KF11 (red star) are indicated. Data represent one of three independent experiments from different donors. (TIF) [file ppat.1004725.s002.tif]

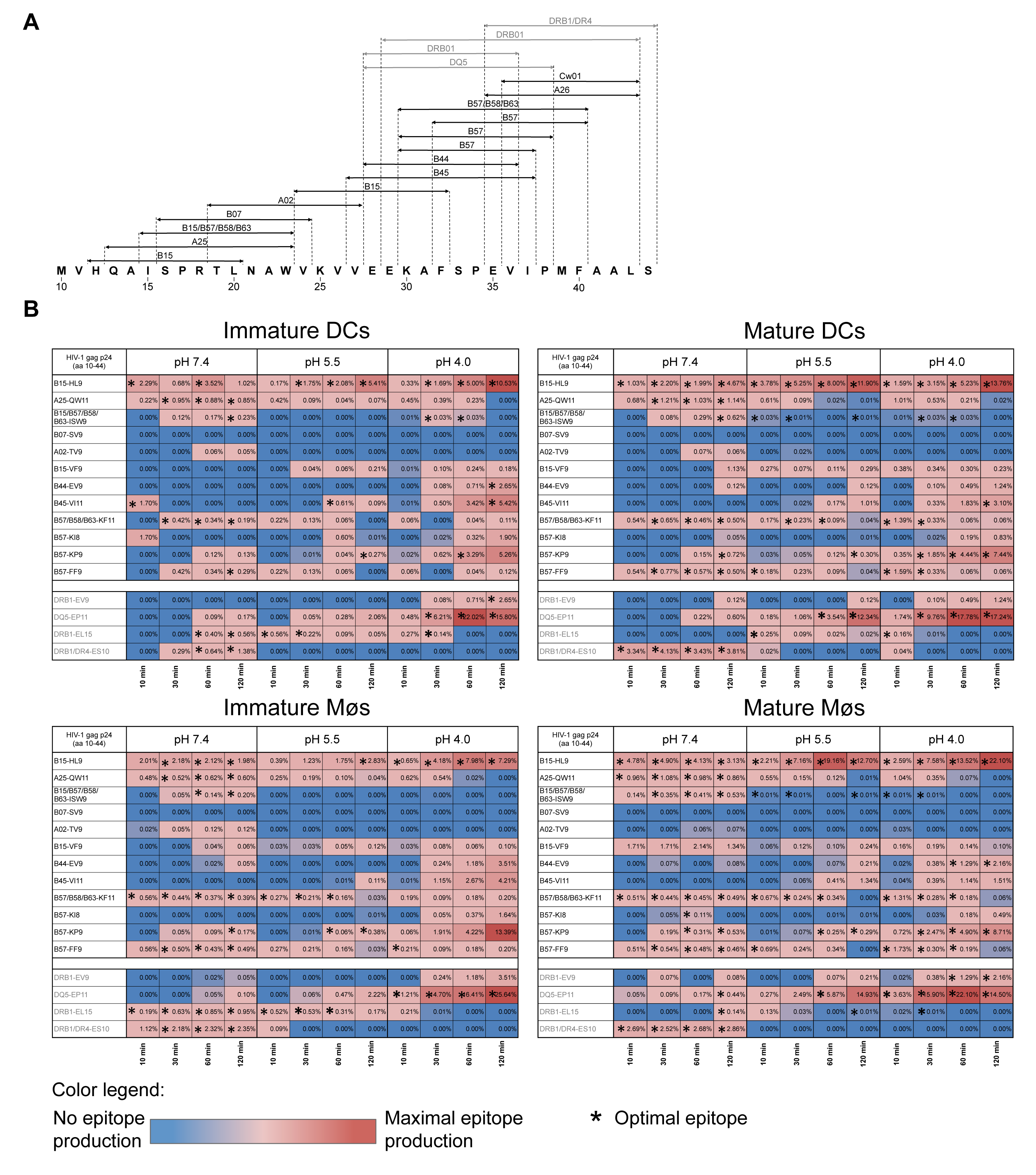

Supplement: S3 Fig — A. The map shows the location of 12 MHC-I epitopes (black arrows) and 4 MHC-II epitopes (gray arrows) within the sequence of Gag p24–35mer (aa 10–44). B. Summary of the relative amount of optimal epitopes and corresponding N-terminal extensions detected by mass spectrometry after 10, 30, 60, and 120 minutes degradation in extracts of immature DCs, mature DCs, immature Møs, mature Møs at pH7.4, pH5.5 and pH4.0. Epitope precursors, defined as peptides with the correct C-terminus and extended by up to three residues at the N-terminus, could be further trimmed in the ER. Numbers represent contribution of optimals and N-extended optimals to the total intensity of all degradation products at each time point. The presence of optimal epitopes is indicated (*). Data represent one of three mass spectrometry analyses from independent experiments. (TIF) [file ppat.1004725.s003.tif]

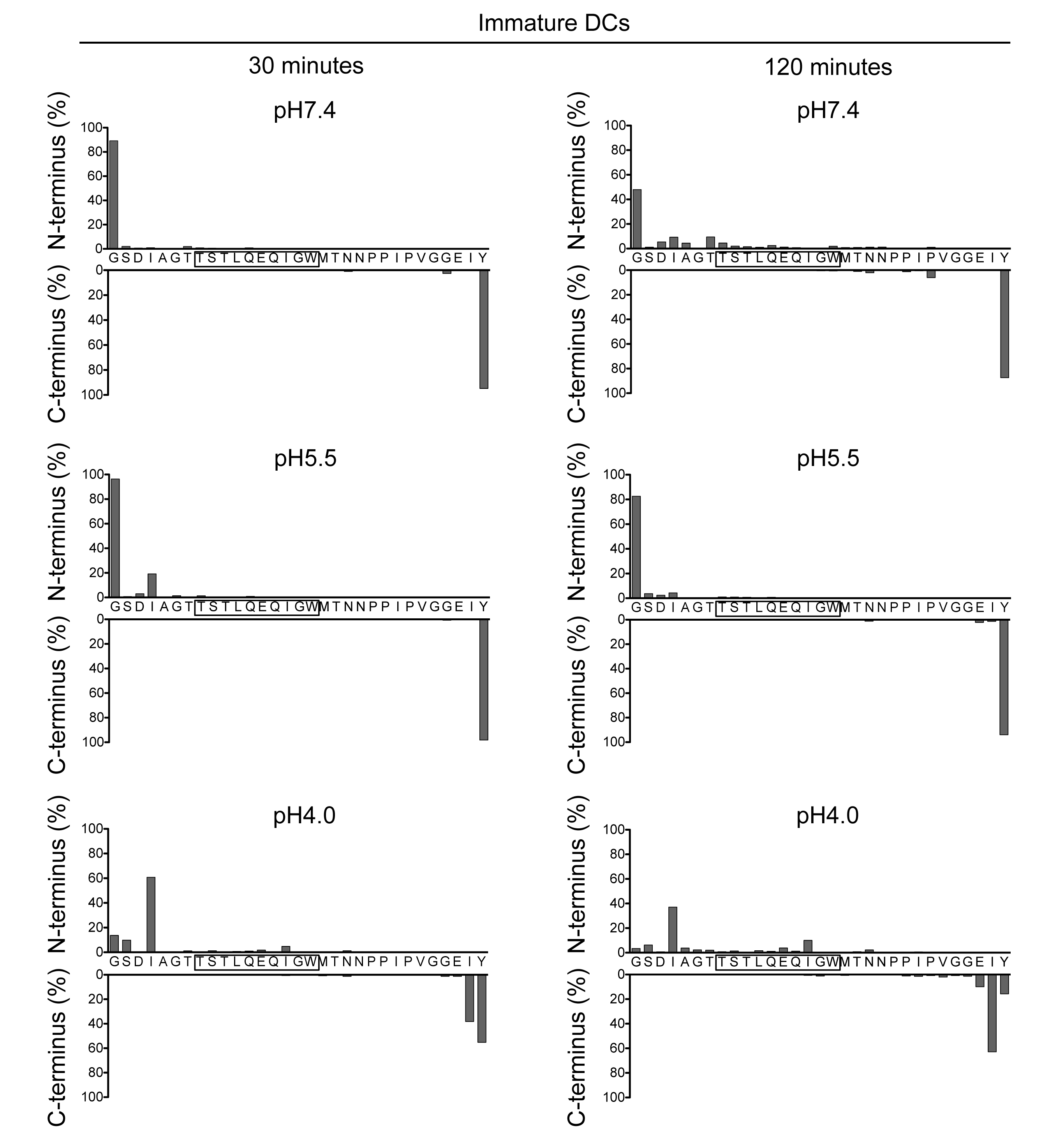

Supplement: S4 Fig — Cleavage patterns of p24–31mer (aa 101–131 in Gag p24) incubated with whole cell extracts from immature DCs for 30 minutes (left panel) or 120 minutes (right panel) at pH7.4, pH5.5, and pH4.0 are shown as the contribution of each cleavage site, presented as cleavage N-terminal or C-terminal to a specific amino acid, to the total intensity of all degradation products. Data are representative of three independent experiments with three different donors. (TIF) [file ppat.1004725.s004.tif]
